# Evaluation of the neuroprotective potential of benzylidene digoxin 15 against oxidative stress in a neuroinflammation models induced by lipopolysaccharide and on neuronal differentiation of hippocampal neural precursor cells

**DOI:** 10.3389/fphar.2025.1537720

**Published:** 2025-03-14

**Authors:** Gilvânia A. Cordeiro, Jessica A. Faria, Leticia Pavan, Israel J. P. Garcia, Eduarda P. F. I. Neves, Gustavo Fernando de Frazao Lima, Hericles M. Campos, Pâmela Y. Ferreira, Paulo C. Ghedini, Elisa M. Kawamoto, Maira C. Lima, José A. F. P. Villar, Ana Maria M. Orellana, Leandro A. Barbosa, Cristoforo Scavone, Jacqueline A. Leite, Hérica L. Santos

**Affiliations:** ^1^ Laboratório de Bioquímica Celular, UFSJ, Universidade Federal de São João del-Rei, Divinópolis, Minas Gerais, Brazil; ^2^ USP, Universidade de São Paulo, São Paulo, Brazil; ^3^ Instituto de Ciências Biológicas, UFG, Universidade Federal de Goiás, Goiânia, Goiás, Brazil; ^4^ Laboratório de Síntese Orgânica e Nanoestruturas, UFSJ, Universidade Federal de São João del-Rei, Divinópolis, Minas Gerais, Brazil

**Keywords:** BD-15, neuroinflammation, cerebellum, prefrontal cortex, oxidative stress, hippocampal neurogenesis

## Abstract

Neuroinflammation, often driven by the overproduction of reactive oxygen species (ROS), plays a crucial role in the pathogenesis of neurodegenerative diseases such as Alzheimer’s and Parkinson’s diseases. The susceptibility of the brain to oxidative stress is attributed to its high metabolic activity and limited antioxidant defense. This study aimed to evaluate the neuroprotective potential of Benzylidene Digoxin 15 (BD-15) following treatment and pretreatment in a lipopolysaccharide (LPS)-induced neuroinflammation model. Additionally, we examined whether BD-15 enhances the generation of neurons from neural progenitor cells (NPCs).Male Wistar rats were used for acute treatment studies and divided into four groups: control (saline), BD-15 (100 μg/kg), LPS (250 μg/kg), and LPS + BD-15 (250 μg/kg + 100 μg/kg). Swiss albino mice were used for chronic pretreatment studies and divided into the following groups: control (saline), BD-15 (0.56 mg/kg), LPS (1 mg/kg), and LPS + BD-15 (1 mg/kg + 0.56 mg/kg). Behavioral changes were assessed using the open field test, and brain tissues were analyzed for oxidative stress markers, including malondialdehyde (MDA), reduced glutathione (GSH), protein carbonylation, catalase (CAT), superoxide dismutase (SOD), and glutathione S-transferase (GST). To assess neurogenesis, primary NPC cultures derived from the hippocampus of newborn Wistar rats were used, which led to reduced locomotor activity and increased oxidative stress, particularly in the cortex, as indicated by elevated MDA levels and reduced GSH levels. BD-15 treatment reversed these effects, notably by restoring GSH levels and reducing protein carbonylation in the cerebellum. Chronic BD-15 treatment in Swiss mice improved oxidative stress markers including MDA, SOD, CAT, and GST. Furthermore, BD-15 exhibits neuroprotective properties by alleviating oxidative stress and motor dysfunction, suggesting its potential as a therapeutic agent for neuroinflammatory disorders. However, BD-15 did not affect NPC cell proliferation, indicating that this cardiotonic steroid did not alter the cell cycle of these progenitor cells.

## 1 Introduction

Neuroinflammation is often associated with neurodegenerative diseases and triggered by numerous factors, including infections, injuries, and autoimmune responses. This process results in increased oxidative stress, increased production of pro-inflammatory cytokines, and alterations in lipid metabolism (Fabelo et al., 2011; Lyman et al., 2014; Shabab et al., 2017; Martín et al., 2010; Wang et al., 2015). The brain, which is particularly susceptible to oxidative stress, is vulnerable owing to its high metabolic activity, extensive use of oxygen, and relatively limited antioxidant defenses (McCubrey et al., 2006; Jeong et al., 2011).

Several neurodegenerative diseases, such as Alzheimer’s and Parkinson’s, are characterized by the overproduction of reactive oxygen species (ROS), leading to oxidative stress and subsequent neuronal damage (McCubrey et al., 2006). The structure of the brain, which includes large quantities of polyunsaturated fatty acids, is a target of oxidative damage, further exacerbating neurodegenerative processes (di Penta et al., 2013; Wang and Michaelis, 2010), contributing to a low regenerative capacity, dysregulation between oxidizing and pro-oxidant agents, impacting the brain as a whole, including motor control, emotional regulation, psychiatric disorders, and cognitive decline (Revuelta et al., 2020; Abg Abd Wahab et al., 2019; Teleanu et al., 2022; Ayoub et al., 2023). Beyond oxidative stress, cognitive disorders and neurodegenerative diseases are also linked to impairments in adult neurogenesis, a process critical for hippocampal plasticity, spatial memory, and learning. Adult neurogenesis involves proliferation, migration, and differentiation of stem cells within the Central Nervous System (CNS). Neural stem cells (NSCs) generate tri-potent neural precursor cells (NPCs), which can differentiate into neurons, astrocytes, or oligodendrocytes in the subgranular zone (SGZ) and the subventricular zone (SVZ) of the brain (Kempermann et al., 2004; Palmer et al., 1997; Kempermann et al., 2004; Palmer et al., 1997). Importantly, damage to these cells such as that induced by lipopolysaccharides (LPS) has been reported to cause significant harm, further highlighting the intricate relationship between neuroinflammation, oxidative stress, and neurodegenerative diseases (Park and Han, 2022).

LPS, a component of the outer membrane of Gram-negative bacteria, is widely used in experimental models to induce neuroinflammation by activating Toll-like receptor 4 (TLR-4) (Abg Abd Wahab et al., 2019; Leow-Dyke et al., 2012). This activation triggers an immune response that leads to the production of pro-inflammatory cytokines and the activation of the nuclear factor kappa B (NF-κB) pathway (Glezer et al., 2003). Given the central role of neuroinflammation in neurodegenerative diseases, researchers have been exploring potential therapeutic strategies to mitigate its effects.

Recent research has shown that cardiac steroids, including derivatives of digoxin, can modulate neuroinflammation by interacting with Na,K-ATPase and by influencing inflammatory signaling pathways (Liu S. J. et al., 2017; Liu T. et al., 2017). The search for new treatments to modulate the inflammatory response in the brain has been an area of intense research. There has been growing interest in the investigation of cardiotonic steroids (CTS), which have been increasingly highlighted as modulators due to studies suggesting that these compounds may have anti-inflammatory and neuroprotective properties, paving the way for the development of new therapeutic strategies for neurological disorders associated with neuroinflammation (Reddy et al., 2020; Schneider et al., 2017; Orellana et al., 2016).

Moreover, CTS have been used to counteract processes triggered by LPS (Garcia et al., 2015; Kinoshita et al., 2014). Some authors have described that low concentrations of CTS, such as ouabain (OUA) (in the nanomolar range), have the ability to interact with Na,K-ATPase (NKA) via the lactone ring at the transmembrane sites TM1-TM2, TM4, TM5-TM6, and TM9-TM10 (Toyoshima et al., 2011; Yatime et al., 2009; Keenan et al., 2005; Shinoda et al., 2009), this interaction enables NKA to function as a signal transducer, activating NF-κB in the central nervous system (CNS). Such enzymatic activation is involved in synaptic processes, neurotransmitter regulation, and neuroprotective activities, playing a crucial role in brain inflammation and neural stem cell proliferation (Orellana et al., 2016; Garcia et al., 2015; De Sá Lima et al., 2013; Garcia et al., 2018; Garcia et al., 2019; Kawamoto et al., 2012). However, at higher concentrations (micromolar range), OUA can exhibit toxicity (Leite et al., 2022; Kinoshita et al., 2022). Interestingly, studies have also demonstrated that OUA affects adult hippocampal neurogenesis by modulating the differentiation of neural precursor cells (NPCs). Specifically, treatment with OUA has been shown to increase the number of mature neurons generated from NPC cultures, suggesting a potential role of this substance in promoting neurogenesis (Maria Orellana et al.).

CTS such as digoxin and OUA bind to α isoforms of NKA (Laursen et al., 2015; Pessôa et al., 2018) which are expressed throughout the body with limited specificity, resulting in a narrow therapeutic index. To address this, new CTS derivatives, such as Benzylidene Digoxin 15 (BD-15), have been explored due to their enhanced binding specificity and low cytotoxic effect (de Oliveira et al., 2021). BD-15 contains non-oxygenated substituents and a hexoxy group attached to the aromatic ring at the para position, along with a methoxy group at the meta position, allowing selective interaction with the α3 NKA isoform via the aromatic ring and amino acid Phe780 (Pessôa et al., 2018). Reports already indicate its neuroprotective potential in ischemia (*in vitro*) and in chronic treatments evaluating the hippocampus and prefrontal cortex of Wistar rats, where an increase activity of the α3 isoform has been observed (de Souza Gonçalves et al., 2019; Parreira et al., 2021).

Thus, the objective of this study is to evaluate the potential effects of BD-15 treatment and pretreatment on neuroinflammation in response to oxidative damage caused by LPS. Given the fundamental role of the brain in cognitive, motor, and emotional functions, this study aims to determine whether BD-15 influences neurogenesis, potentially offering a novel therapeutic approach for neuroinflammation-related neurological disorders.

## 2 Materials and methods

### 2.1 Acute post-treatment of wistar rats

Wistar rats were used as model for acute post-treatment of BD-15 and challenged with LPS. The experiments conducted in this study followed the Ethical Principles of Animal Experimentation adopted by the National Council for the Control of Animal Experimentation (CONCEA) and were approved by the Animal Use Ethics Committee (CEUA) of the Federal University of São João del-Rei (protocol no. 7812090522.

Male Wistar rats, 3 months of age, were kept under a 12-hour light-dark cycle with free access to food and water. The treatments were conducted as follows:

1. CTR (Control Group): Animals received only saline solution intraperitoneally (i.p.), followed by another saline injection i.p. after 20 min.
2. BD-15 group: Animals received an i.p injection of BD-15 at a concentration of 100 μg/kg (nanomolar levels), followed by a saline injection after 20 min.
3. LPS Group: Animals first received an i.p. saline injection, followed by LPS at a concentration of 250 μg/kg after 20 min.
4. LPS + BD-15 Group: Animals first received an i.p injection of LPS (250 μg/kg) and, after 20 min, an injection of BD-15 at a concentration of 100 μg/kg.

After 2 h of treatment, the animals underwent the open field behavioral test and were then euthanized by decapitation (Garcia et al., 2019). Their brains were immediately removed and placed on ice before being stored at −80°C for subsequent biochemical analyses.

### 2.2 Chronic pretreatment of swiss mice

Swiss mice were used for a long-term treatment of BD-15 (3 consecutive days) and LPS model (1 day). All the experimental procedures were approved by the Ethics Committee on Animal Use of the Federal University of Goiás (protocol CEUA/UFG no. 014/21). Two-month-old Swiss albino mice were obtained from the Central Bioterium of the Federal University of Goiás (UFG) and maintained in the Department of Physiology and Pharmacology (DciF/Dfar) at the Institute of Biological Sciences (ICB/UFG). The animals were kept at a controlled temperature of 22°C ± 2°C with a 12-hour light/dark cycle and had free access to food and water. Mice were divided into four groups of seven animals each.

1. CTR (Control Group): Animals received only saline solution intraperitoneally (i.p.).
2. BD-15 group: Animals received an i.p injection of BD-15 at a concentration of 0.56 mg/kg
3. LPS Group: Animals first received an i.p. of LPS at a concentration of 1 mg/kg (Yang et al., 2020)
4. LPS + BD-15 group: Animals first received an i.p injection of LPS (1 mg/kg) and after an injection of BD-15 at a concentration of 0.56 mg/kg.

**SCHEME 1:**
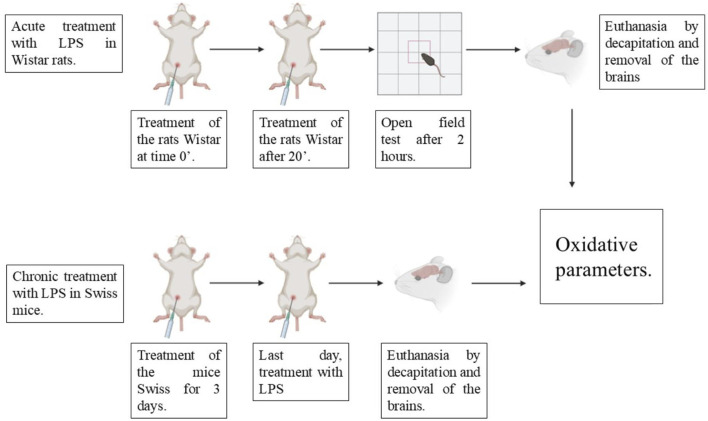
Therapeutic protocol for the induction of acute and chronic neuroinflammation by LPS.

I.p injections of saline solution or BD-15 were administered for three consecutive days. On the last day, 1 hour after treatment, the animals were challenged with LPS by i.p injection. After 24 h, the animals were euthanized to remove their cortex and cerebellum for biochemical analysis (Scheme 1).

### 2.3 Preparation of tissue samples

In summary, the cerebellar regions (cortex and cerebellum) of animals were individually homogenized on ice using a Potter-Elvehjem homogenizer. Homogenization was performed using 10 strokes in a membrane preparation buffer containing 10 mM Tris (pH 7.4), 320 mM sucrose, 0.5 mM EDTA, and 1 mM magnesium chloride, supplemented with protease inhibitors (2 μg/mL leupeptin, 2 μg/mL antipain, and 3 mM sodium orthovanadate). The total supernatants were considered homogenized tissue and were used to measure biochemical markers of oxidative stress. Additionally, the supernatants were ultracentrifuged (52,000 × g, 4°C, for 1 h) and the pellets were resuspended in membrane preparation buffer. The protein content of the cortex and cerebellum homogenates was determined using the Bradford method, with bovine serum albumin as a standard for Bradford method (Bradford, 1976). All biochemical analyses were performed in triplicates.

### 2.4 Primary culture of neural stem cells—Neurospheres

Neonatal Wistar rats, at the postnatal age of P0-P4, from the Central Animal Facility of the Institute of Biomedical Sciences, University of São Paulo, were euthanized by decapitation, and we dissected the hippocampus of both hemispheres. A pool of hippocampus from four animals was used in each cell culture. All the assays were performed with primary culture without passages to reduce variability (The experiments were performed according to the ARRIVE guidelines and under the norms of the Ethical Committee for Animal Research of the Institute of Biomedical Sciences (CEUA/ICB/USP, protocol no. 74/2017).

The protocol to obtain the neurospheres followed by Okun et al. (2010). Briefly, hippocampi were placed in cold Hanks' Balanced Saline Solution or HBSS (53.33 mM KCl, 4.41 mM KH_2_PO_4_, 1.37 M NaCl, 3.36 mM Na_2_HPO_4_ and 55.56 mM D-glucose). The HBSS solution was aspirated, and the sections were incubated for 30 min in Trypsin solution (0.05%) at 37°C. After incubation, trypsin was removed. The tissues were washed once with HBSS at 37°C. After that, cell culture medium 1 was added [Medium 1: DMEM/F12 (Dulbecco’s modified Eagle’s medium/Ham’s F-12 medium) supplemented with B27, epidermal growth factor (EGF), Fibroblast Growth Factor (bFGF) (both at a concentration of 20 ng/mL) and Penicillin/Streptomycin at a final concentration of 50 U/mL]. Tissue was mechanically dissociated in medium 1, plated, and kept in this medium for 7 days, 37°C, 5% CO_2_, during which neurospheres developed. Two different groups were analyzed: one received artificial cerebrospinal fluid or aCSF (NaCl 111 mM; KCl 4mM; NaH_2_PO_4_ 1 mM; NaHCO_3_ 25 mM; CaCl_2_ 2H_2_O 1.5 mM; Glucose 10 mM; HEPES 20 mM; p.H 7.4); and received BD15. Treatment was added to the medium immediately after cells were plated in the same volume. Artificial cerebrospinal fluid (aCSF) was used to prepare the BD15 solution. In both bottles, 8 × 10^4^ cells were plated. The hippocampus of three to four neonates comprises a bottle of cell culture. The neurospheres were dissociated after 7 days, according to Walker and Kempermann (2014). Briefly, the cell culture medium containing the neurospheres was transferred to a 15 mL tube and centrifuged at 300× *g* for 5 min. The supernatant was discarded, and the cells were resuspended with trypsin-EDTA (0.05%) for 3 min at room temperature (RT). Trypsin-inhibitor (0.125 mg/mL) with 0.01 mg/mL of DNAse I was added to stop the reaction. Cells were centrifuged 300× *g* for 5 min at room temperature. The supernatant was removed, and cells were washed twice with HBSS 37 °C. The medium was added, and cells were triturated up and down with a pipette to dissociate neurospheres. Cells were reseeded at a density of 1 × 10^4^ cells/cm^2^ in the appropriate well or flask, according to the assay to be performed.

### 2.5 Open field test

The animals were placed in an acrylic box (70 cm × 70 cm × 40 cm) with one transparent side and evaluated for 5 min. The floor of the box is divided into twenty-five quadrants. Animals were placed in the same starting quadrant. The following parameters were measured: number of quadrant crossings, number of central quadrant crossings, rearing (number of times the animal was reared), and grooming duration. These measurements were used to assess the locomotor and exploratory activities of the animals. A higher number of visits to the central quadrant indicates lower anxiety levels.

### 2.6 Biochemical parameters

#### 2.6.1 Determination of thiobarbituric acid reactive substances (TBARS)

This methodology was performed as described by Buege (1978) with adaptations, in which 50 μL of the soluble fraction from [different brain structures originating from rats (cerebellum and hippocampus) and mice (hippocampus, cortex, and hypothalamus)] the cortex and cerebellum were transferred to a conical tube, and the final volume was adjusted to 200 µL with 50 mM sodium phosphate buffer (pH 7.4). Next, 200 µL of 12% trichloroacetic acid and 400 µL of 1% thiobarbituric acid were added to the tube. The mixture was shaken vigorously and incubated in a water bath at 95°C for 30 min. After incubation, the tubes were placed in an ice bath for 15 min and centrifuged at 10,000 g for 5 min. The absorbance was measured at 532 nm using a spectrophotometer (Buege and Aust, 1978).

#### 2.6.2 Reduced glutathione (GSH)

The assay was based on the reaction between the thiol group of GSH and 5.5′-dithio-bis-(2-nitrobenzoic acid) (DTNB), forming a yellow-colored product. Twenty-five microliters of homogenized tissue was added to Tris buffer (1M, pH 8.0) containing 10 mM DTNB. The reaction mixture was incubated for 15 min, and the absorbance was immediately measured at 412 nm. The results were expressed as µg of GSH per milligram of protein (Garcia et al., 2019).

#### 2.6.3 Protein carbonyl content assay

The carbonyl content in brain homogenates was analyzed using the method of Levine ad collaborators (Levine et al., 1990), with adaptations. The assay is based on the spectrophotometric detection of the reaction with 2,4-dinitrophenylhydrazine. The data were expressed in nmol of carbonyl groups/mg of protein.

#### 2.6.4 Superoxide dismutase (SOD) activity

The sperm SOD Activity were spectrophotometrically determined. The principle of this method is the ability of superoxide dismutase enzyme to inhibit the autoxidation of epinephrine. The sperm were incubated with epinephrine bitartrate 60 mmol/L, and the sample color intensity was masured at 480 nm, according to the method of Misra and Fridovich (1972) with some modifications. The enzymatic activity is expressed in units (U) of SOD/mg of protein.

### 2.7 Catalase (CAT) activity

The sperm CAT activity was spectrophotometrically determined by the H2O2 decomposition at 240 nm according to the method described by Aebi (1984) with some modifications. The sperm sample were incubated with 86 mmol/L H2O2 and sodium phosphate buffer (pH 7.0). The enzymatic activity is expressed in units (U) of CAT/mg of protein. One U of enzyme thus decomposed 1 µmol of H2O2/min at pH 7.0 at 25°C.

## 3 Cytotoxicity assays in NPCs cells

### 3.1 Protocol for cell viability assay: Formazan color reduction (MTT)

The MTT and LDH assay were carried out using a primary culture of neural precursor cells from the hippocampus of neonatal rats. The cultures were incubated for 4 days in a medium conducive to proliferation and grew floating, generating neurospheres. This colorimetric assay is based on the reduction of a yellow tetrazolium salt (3-[4,5-dimethylthiazol-2-yl]-2,5-diphenyltetrazolium bromide or MTT) (Selvakumaran and Jell, 2005) to purple formazan crystals by metabolically active cells, measuring the mitochondrial activity and consequent viability of the cells. The viability of cells submitted to the different types of treatment was determined by adding 12 mM MTT to the DMEM/F12 culture medium (with Penicillin/Streptomycin 50 U/mL). The cells had adhered to the plates, the treatments were carried out in the 48-well plates and a time delay of 24 h. The supernatant containing the treatments was removed and used to carry out the LDH assay, while culture medium with diluted MTT was added to each treated well culture medium with diluted MTT. The plates were incubated at 37°C for 2. The plates were incubated at 37°C for 2 h, after which time the culture medium was removed and the dark crystals dissolved by adding DMSO. A volume of 50 μL was transferred to a 96-well plate and the absorbance was measured by a microplate reader at a wavelength of 570 nm. Two wells of the plate received 0.1% DMSO as a negative control for the assay.

### 3.2 Lactate dehydrogenase (LDH) release

The activity of the enzyme lactate dehydrogenase (LDH) were measured using the kit CytoTox 96 Non-Radioactive Cytotoxicity Assay (Promega). This cytosolic enzyme is released from cells due to the process of membrane lysis, thus LDH converts lactate into pyruvate by reducing NAD + to NADH. The level of formazan formation is directly proportional to the amount of LDH released into the medium. After the cells had adhered to the plates, the treatments were carried out in the 48-well plates. After 24 h, 50 μL of lysis buffer from the kit was added to two wells. Buffer from the kit was added to two wells as a positive control representing the maximum LDH release. The plate was incubated for 45 min at 37°C. The supernatant from each well was removed and transferred to a 96-well plate. The same volume of reaction buffer from the kit and the plate were incubated at room temperature and protected from light for 45 min, after which 50 mL of stop solution was added. The staining was read using a spectrophotometer at a wavelength of 490 nm. Two wells of the plate were used as the negative control of the assay, containing only culture medium serving as the background of the reading.

## 4 NPCs cell proliferation

The cell proliferation assay by cell count was carried out following the protocol established by Baumann et al. (2014), which consists in analyzing the number of neurospheres in each treatment over a given period. This test was carried out to measure the ability of the neural precursor cell to divide and generate new cells (neurospheres) over time. Initially, photos were taken of the untreated neurospheres at 4 and 7 days. After the seventh day without treatment, the neurospheres were treated with decreasing concentrations of BD-15: 1 μM, 500 nM, 250 nM, 150 nM, 50 nM and the control DMSO 0.1%. The day after treatment, the number of neurospheres in each treatment well was counted. On the third day, in addition to counting the neurospheres, more DMEM/F12 medium was added with the growth factors so that the neurospheres would not die (as they last an average of 4 days). On the seventh day and the last day of the test, the count was carried out again. The count under a light microscope with a manual counter.

## 5 NPCs cell differentiation

To verify the effect of BD-15 on the cell cycle and its ability to influence differentiation, cells were divided into four groups. In control group (i), characterized by “CTR/no treatment”, the cells remained untreated with BD-15; in group (ii) “CTR/BD15 250 nM”, the previously untreated cells were subsequently plated and treated with BD-15 250 nM, making it possible to analyze the interference of BD-15 in proliferation; in group (iii) “BD15 250 nM/no treatment”, cells previously treated with BD-15 250 nM were plated without treatment, and it was possible to analyze whether there would be differentiation by removing BD-15; and in group (iv) “BD15 250 nM/BD15 250 nM”, the cells remained treated with BD-15 250 nM throughout the experimentation and with this, it would be possible to analyze cell behavior with BD-15 100% of the time. For the cells that were dissociated, they were plated in a 24-well plate at a concentration of 30,000 cells per well, on a glass coverslip previously treated with 5% PEI (Polyethyleneimine). During dissociation, to check differentiation, the culture medium in which the cells were kept was medium 2. Medium 2 contains: DMEM:F12 supplemented with B27, NGF (nerve growth factor, 20 ng/mL) and Penicillin/Streptomycin at a final concentration of 50 U/mL, in the absence of the growth factors EGF and bFGF, responsible for maintaining the cells in an undifferentiated state, as described in Schramm and Schulte (2014).

## 6 Statistical analysis

*In vivo* Studies: Statistical analyses and graph generation were performed using GraphPad Prism, version 8. Data are expressed as mean ± standard error of the mean (SEM). Normality between groups was assessed using the Kolmogorov-Smirnov test. Differences between groups were analyzed using one-way analysis of variance (ANOVA) followed by Tukey’s *post hoc* test. Statistical significance was set at p < 0.05. Results from Western blot and immunofluorescence assays were analyzed as optical density by the program ImageJ (National Institutes of Health, United States).

NPCs experiments: Immunofluorescence quantification was studied in three ways: (i) to know the percentage of each cell population. We used the cell counter plugin, (ii) to measure fluorescence intensity, the fluorescent beads as an internal standard, and (iii) to analyze neuronal dendritic area. We also used the Sholl Analysis plugin from ImageJ (National Institute of Health, United States).

## 7 Results

### 7.1 Behavioral test

Following ours previously studies with LPS as a model of neuroinflammation (Kinoshita et al., 2014) we evaluated the effect of acute post-treatment with BD-15 on locomotor activity in the open field test.

**FIGURE 1:**
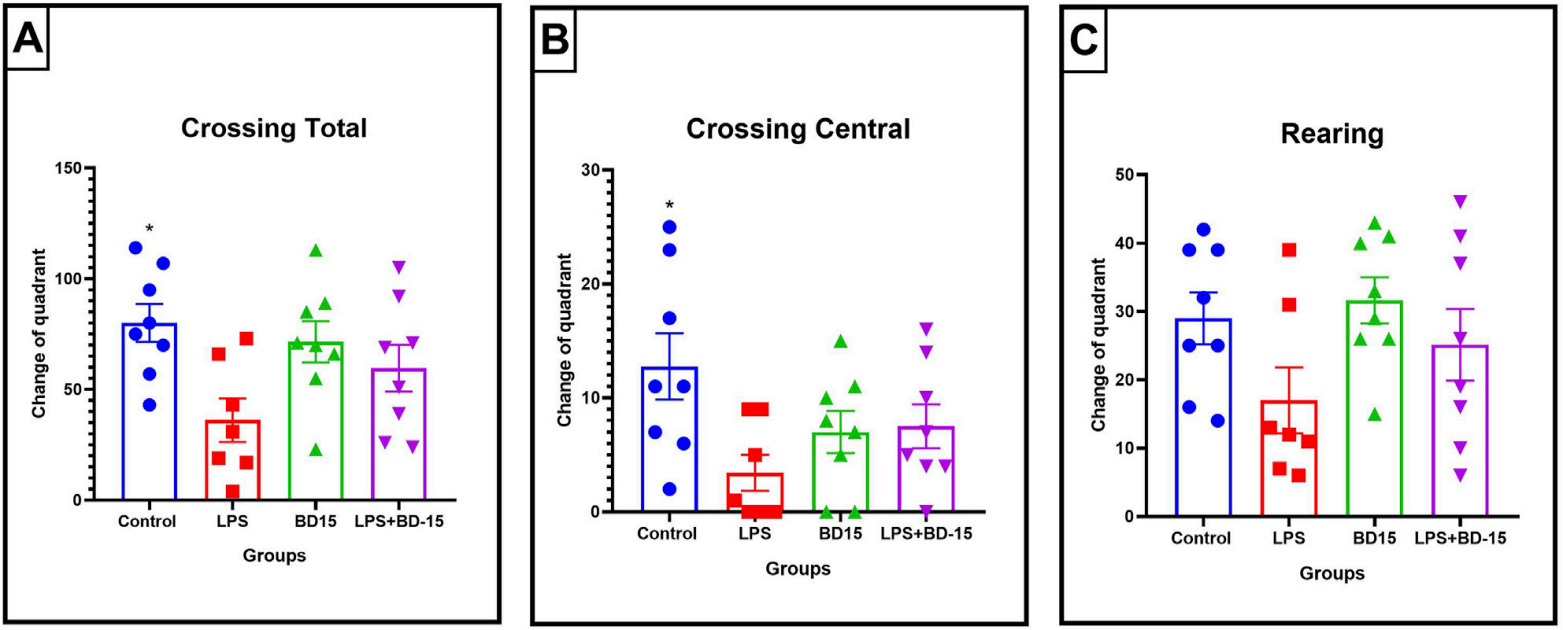
Parameters evaluated through the open field test. The data are expressed as Mean ± SEM. Total crossings (quadrant changes) (control: 80.13 ± 8.591; LPS: 36.13 ± 9.78; BD-15: 71.50 ± 9.324; LPS + BD-15: 59.63 ± 10.54) **(A)**; Central crossings (central quadrant changes) (control: 12.75 ± 2.908; LPS: 3.429 ± 1.587; BD-15: 7.00 ± 1.852; LPS + BD-15: 7.5 ± 1.927) **(B)**; Rearing time in seconds (control: 7.75 ± 2.795; LPS: 9.714 ± 3.926; BD-15: 7.375 ± 3.00; LPS + BD-15: 6.375 ± 2.449) **(C)**. The data were subjected to ANOVA, and *p < 0.05 was adopted as the significance level.

Here, after a 2-hour total treatment, our results demonstrated that the LPS group (36.13 ± 9.78 quadrant crossings) exhibited a significant 55% reduction in locomotor activity compared to the control group (80.13 ± 8.59 quadrant crossings), according to total crossing parameters (*p < 0.05) (Figure 1A). Furthermore, a 73% reduction in the number of visits to the central quadrants was observed in the LPS group (control: 12.75 ± 2.91; LPS: 3.43 ± 1.59) (Figure 1B), indicating a likely motor dysfunction since no significant change was found in rearing behavior (Figure 1C). BD-15 was not able to prevent the locomotor effect provoked by LPS.

However, BD-15 did not induce any significant changes in any of the evaluated parameters when compared to the other groups, including the control group. In other words, BD-15 did not cause any behavioral impairments. Given the motor and locomotor alterations observed in LPS-treated animals, we then analyzed oxidative parameters in the cortex and cerebellum to gain a deeper understanding of these findings. These brain regions were specifically chosen due to their critical role in motor function.

### 7.2 Effect of BD-15 on oxidative stress

**FIGURE 2:**
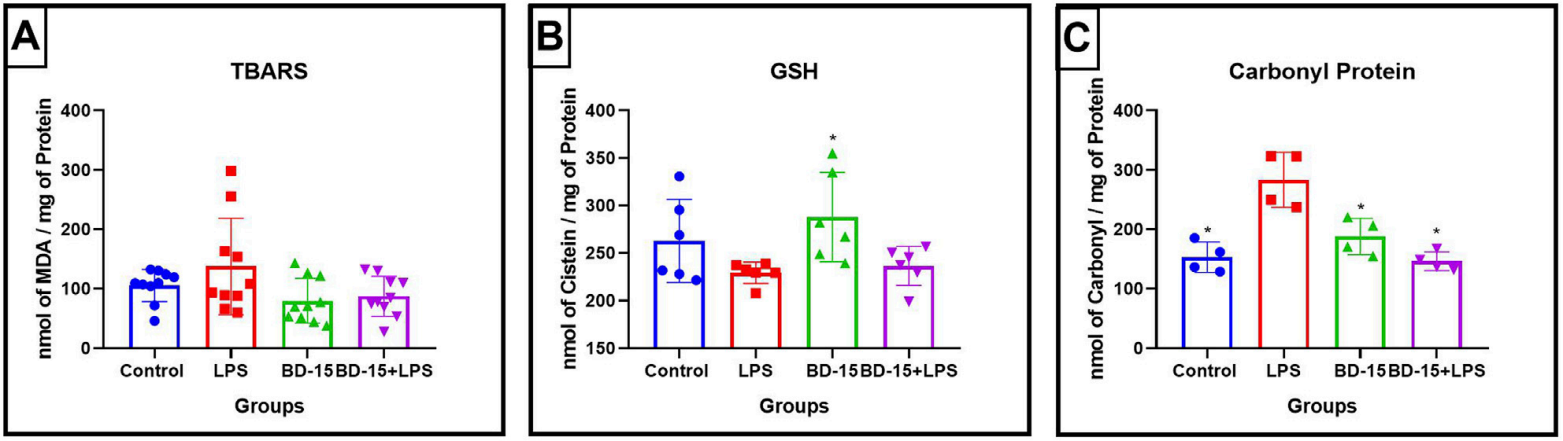
Evaluation of oxidative parameters in the cerebellum of Wistar rats. Data are presented as mean ± SEM MDA levels levels (Control: 105.6 ± 27.03; LPS: 137.7 ± 81.16; BD-15: 79.88 ± 37.52; LPS + BD-15: 87.35 ± 33.59) **(A)**. Data are presented as mean ± SEM GSH levels (Control: 262.8 ± 47.75; LPS: 229.2 ± 11.19; 228 ± 46.98; LPS + BD-15: 236.5 ± 20.67) **(B)**, and data are presented as mean ± SEM carbonyl protein levels (Control: 153.2 ± 25.65; LPS: 283.3 ± 46.05; 187.9 ± 30.56; 146.4 ± 15.73) **(C)** of rats treated with LPS and BD-15. Statistical analysis was performed using one-way ANOVA. Adopting of *p < 0.05, **p < 0.005, ***p = 0.0001, for comparisons with the LPS group significant difference for the LPS group compared to the others.

In the same acute protocol, we analyzed oxidative stress markers in the cerebellum of rats challenged with LPS by measuring malondialdehyde (MDA) and reduced glutathione (GSH) levels. Our findings revealed that LPS administration did not significantly alter these parameters compared to the control (CTR) group, nor did post-treatment with BD-15 induce any changes (Figures 2A, B). However, when assessing protein carbonylation, a marker of oxidative protein damage, we observed a significant increase in the LPS-treated group, which was effectively reversed by post-treatment with BD-15 (Figure 2C).

**FIGURE 3:**
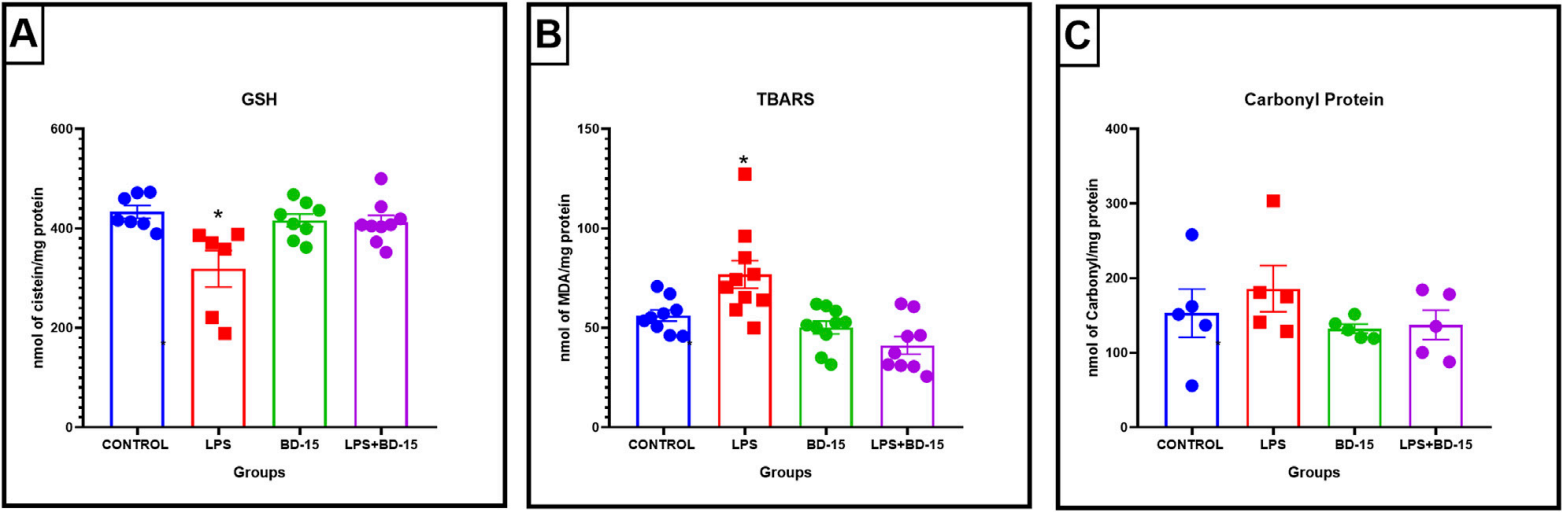
Evaluation of oxidative parameters in the cortex of Wistar rats. Data are presented as mean ± SEM MDA levels (control: 56.53 ± 2,859; LPS: 76.89 ± 6,962; BD-15: 50.22 ± 3,205; LPS + BD-15: 41.27 ± 4,442 **(A)**. Data are presented as mean ± SEM GSH levels (control: 433.5 ± 12.86; LPS: 318.9 ± 36.56; BD-15: 416.4 ± 13.02; LPS + BD-15: 412.5 ± 14.00 **(B)**, and data are presented as mean ± SEM carbonyl protein levels (control: 153.1 ± 32.30; LPS: 185.9 ± 31.08; BD-15: 132.2 ± 6,058; LPS + BD-15: 137.4 ± 19.63) **(C)** of rats treated with LPS and BD-15. Statistical analysis was performed using one-way ANOVA. Adopting *p < 0.05 significant difference for the LPS group compared to the others.

A similar analysis in the prefrontal cortex showed a statistically significant difference in MDA and GSH levels in the LPS-treated groups compared to the other treatment groups (Figures 3A, B). However, unlike in the cerebellum, no significant changes were detected in protein carbonylation levels in this brain region (Figure 3C). These findings suggest that while LPS induces oxidative stress in the prefrontal cortex, its impact on protein oxidation appears to be more prominent in the cerebellum.

Given the outcomes observed in the acute post-treatment protocol with Wistar rats, along with previous findings from chronic treatment studies using isolated BD-15 administration (Parreira et al., 2021), we hypothesized that a chronic or preventive BD-15 treatment followed by LPS exposure could yield more definitive protective effects. This approach could provide a clearer understanding of BD-15’s potential neuroprotective role in mitigating oxidative stress and neuroinflammation.

To further refine our investigation, we considered the importance of employing both rat and mouse models in pharmacological studies. Research using both species allows for a more comprehensive assessment of species-specific metabolism and toxicity profiles, which are particularly relevant in the context of neuroinflammation (Chiu et al., 2016). This dual-species approach not only helps identify potential safety concerns but also enhances the translational relevance of preclinical findings, bringing experimental research one step closer to human applications (Marklund and Hillered, 2011).

### 7.3 Chronic pretreatment with BD-15 in swiss mice

**FIGURE 4:**
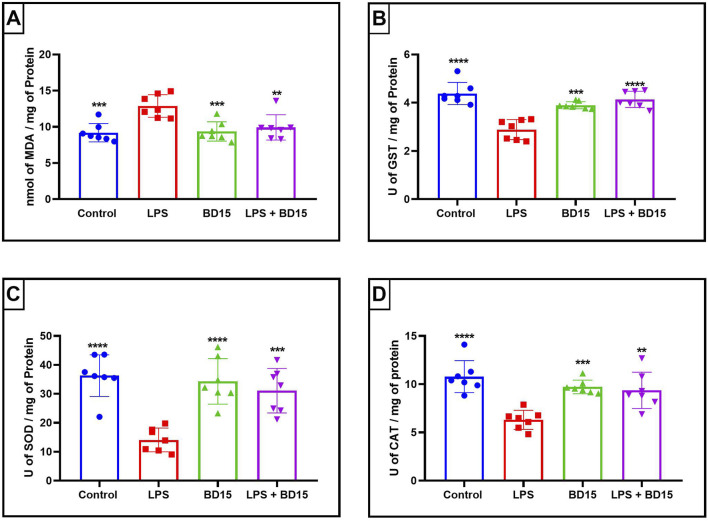
Biochemical parameters of the cortex of Swiss mice treated with LPS and/or BD-15. Data are presented as mean ± SEM MDA level (Control: 9,194 ± 1,272; 12.89 ± 1,564; 9,358 ± 1,334; 9,928 ± 1,752) **(A)**. Data are presented as mean ± SEM GST activity (4,382 ± 0,4586; 2,887 ± 0,4134; 3,895 ± 0,1494; 4,139 ± 0,3386) **(B)**. Data are presented as mean ± SEM SOD activity (36.30 ± 7,183; 14.08 ± 4,103; 34.32 ± 7,888; 31.10 ± 7,687) **(C)** and data rea presented as mean ± SEM CAT activity (10.78 ± 1,657; 6,310 ± 0,9852; 9,714 ± 0,7078; 9,363 ± 1,876) **(D)**. Statistical analysis was performed using one-way ANOVA with significance levels of *p < 0.05, **p < 0.005, ***p = 0.0001, and ****p < 0.0001 for comparisons with the LPS group.

**FIGURE 5:**
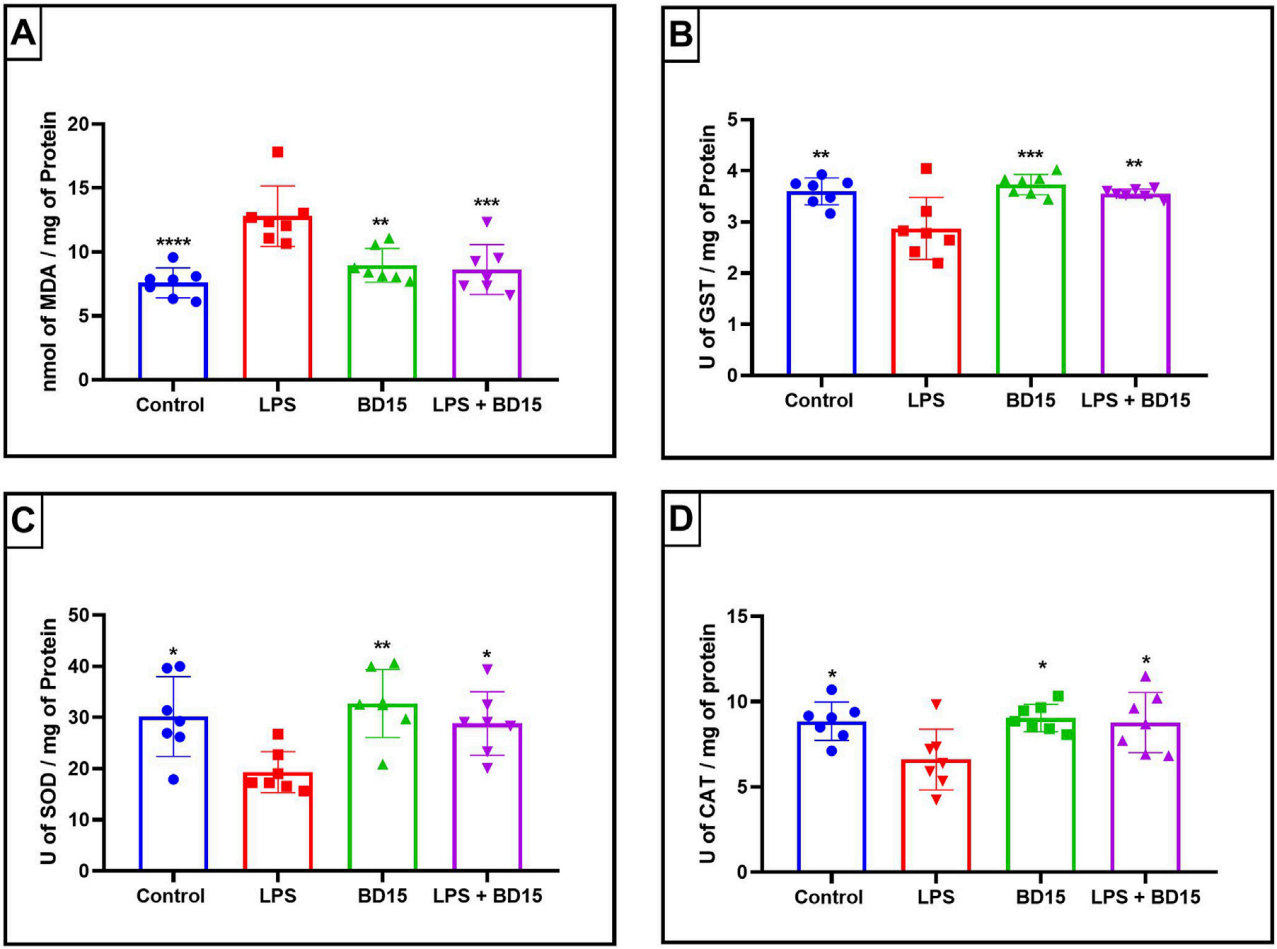
Biochemical parameters of the hippocampus of Swiss mice treated with LPS and/or BD-15. Data are presented as mean ± SEM MDA levels (Control: 7,585 ± 1,169; 12.8 ± 2,362; 8,959 ± 1,324; 8,622 ± 1,945) **(A)**. Data are presented as mean ± SEM GST activity (3,599 ± 0,2622; 2,874 ± 0,6062; 3,729 ± 0.2; 3,557 ± 0,0876) **(B)**. Data are presented as mean ± SEM SOD activity (30.19 ± 7,795; 19.31 ± 4,007; 32.74 ± 6,650; 28.81 ± 6,209) **(C)** and data rea presented as mean ± SEM CAT activity (8,855 ± 1,129; 6,617 ± 1,781; 9,045 ± 0,8020; 8,782 ± 1,760) **(D)**. Statistical analysis was performed using one-way ANOVA with significance levels of *p < 0.05, **p < 0.005, ***p = 0.0001, and ****p < 0.0001 for comparisons with the LPS group.

**FIGURE 6:**
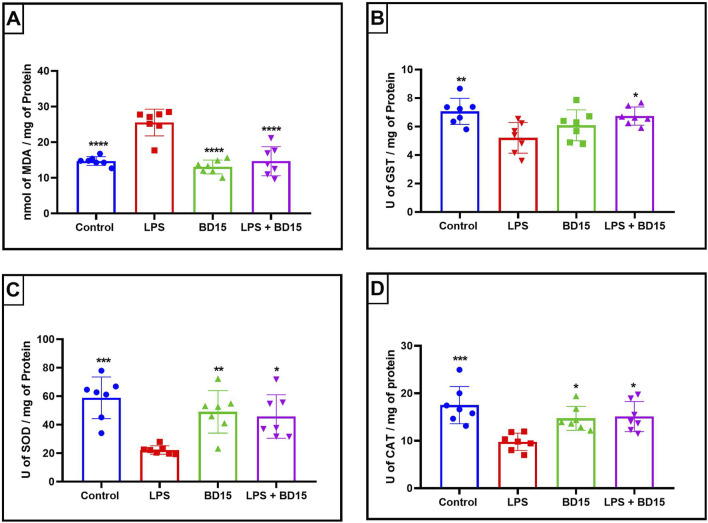
Biochemical parameters of the hypothalamus of Swiss mice treated with LPS and/or BD-15. Data are presented as mean ± SEM MDA levels (Control: 14.71 ± 1,241; 25.53 ± 3,734; 13.06 ± 1,929; 14.67 ± 4,088) **(A)**. Data are presented as mean ± SEM GST activity (7,061 ± 0,9181; 5,201 ± 1,076; 6,087 ± 1,083; 6,734 ± 0,6363) **(B)**. Data are presented as mean ± SEM SOD activity (58.96 ± 14.63; 22.26 ± 2,936; 49.03 ± 14.94; 45.78 ± 15.24) **(C)** and data rea presented as mean ± SEM CAT activity (17.51 ± 3,932; 9,783 ± 1,830; 14.73 ± 2,522; 15.10 ± 3,167) **(D)**. Statistical analysis was performed using one-way ANOVA with significance levels of *p < 0.05, **p < 0.005, ***p = 0.0001, and ****p < 0.0001 for comparisons with the LPS group.

Swiss albino mice were pretreated with BD-15 for three consecutive days, followed by exposure to LPS. The results revealed significant biochemical changes across the evaluated brain regions: the cortex (Figure 4), the hippocampus (Figure 5), and the hypothalamus (Figure 6).

Except for the hypothalamus, which showed no significant difference between the LPS-treated group and the BD-15 pretreated group in the assessment of glutathione S-transferase (GST) activity (Figure 5B), all other areas evaluated exhibited statistically significant differences in MDA levels, GST activity, superoxide dismutase (SOD) activity, and catalase (CAT) activity between the LPS group and the other treatments. These results indicate that BD-15 pretreated has the potential to reverse LPS-induced oxidative damage when used chronically and preventively.

Since we visualized the BD-15 neuroprotection on mice and rats brain sections, it is important to understand which mechanisms could be involved. One mechanism that could cause this effect is neurogenesis, that is already observed for ouabain treatment (Maria Orellana et al.).

### 7.4 Treatment of hippocampal NPCs with different concentrations of BD-15 in cell viability

**FIGURE 7:**
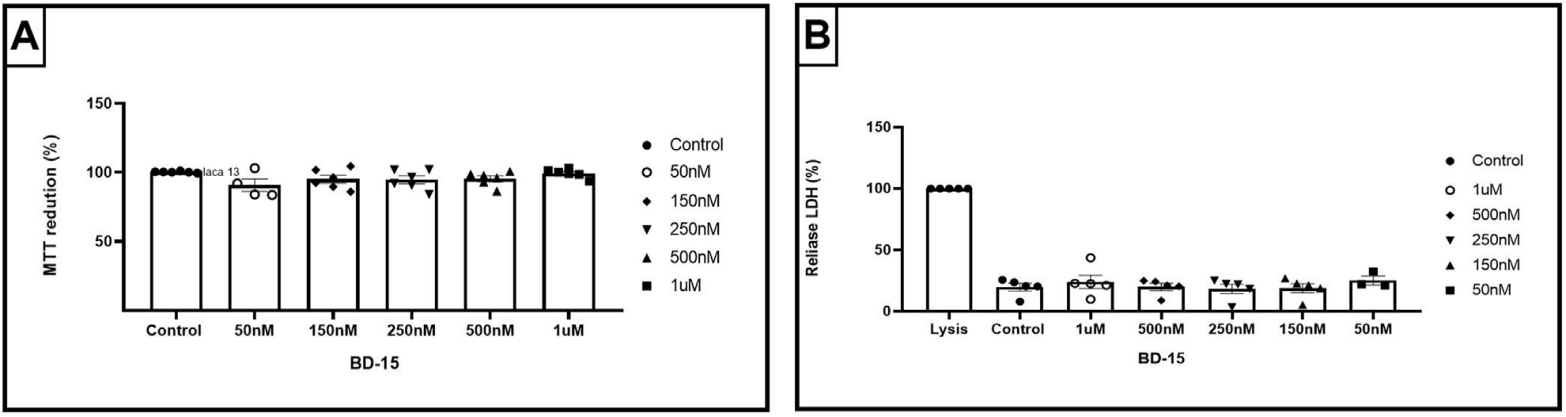
Cytotoxicity parameters of BD-15 by MTT and LDH assays. **(A)** Data are presented as mean ± SEM (100.2 ± 0.215; 99.18 ± 1,318; 95.31 ± 2,088; 94.54 ± 2,863; 95.02 ± 2,906; 90.64 ± 4.52). **(B)** Data are presented as mean ± SEM (100 ± 0; 19.78 ± 3,097; 25.28 ± 3,680; 19.01 ± 3,687; 28.35 ± 3,839; 20.02 ± 2,914; 24.11 ± 5,470). Statistical analysis was performed using one-way ANOVA with significance levels of *p < 0.05.

Our results showed no significant differences (p = 0.1416) when comparing the concentrations tested with the control (diluent vehicle of BD-15) DMSO 0.1%, concluding that BD-15 does not alter the viability by the MTT method. To complement the MTT tests, we used the same concentrations of BD-15; we could observe in the LDH assays that BD-15 also did not cause cell lysis (p = 0.7991) at any of the concentrations tested. Figure 7 shows the cell lysis control, identified by Lysis, and the positive control DMSO 0.1% compared to the concentrations of BD-15 used. The Lysis treatment refers to the lysis buffer, where all the cells treated with this buffer are lysed 100%. Since we did not observed cytotoxic effect for NPCs we assessed the effect of BD-15 on proliferation and differecntiation.

### 7.5 Treatment of hippocampal NPCs with different concentrations of BD-15 in cell proliferation and differentiation

**FIGURE 8:**
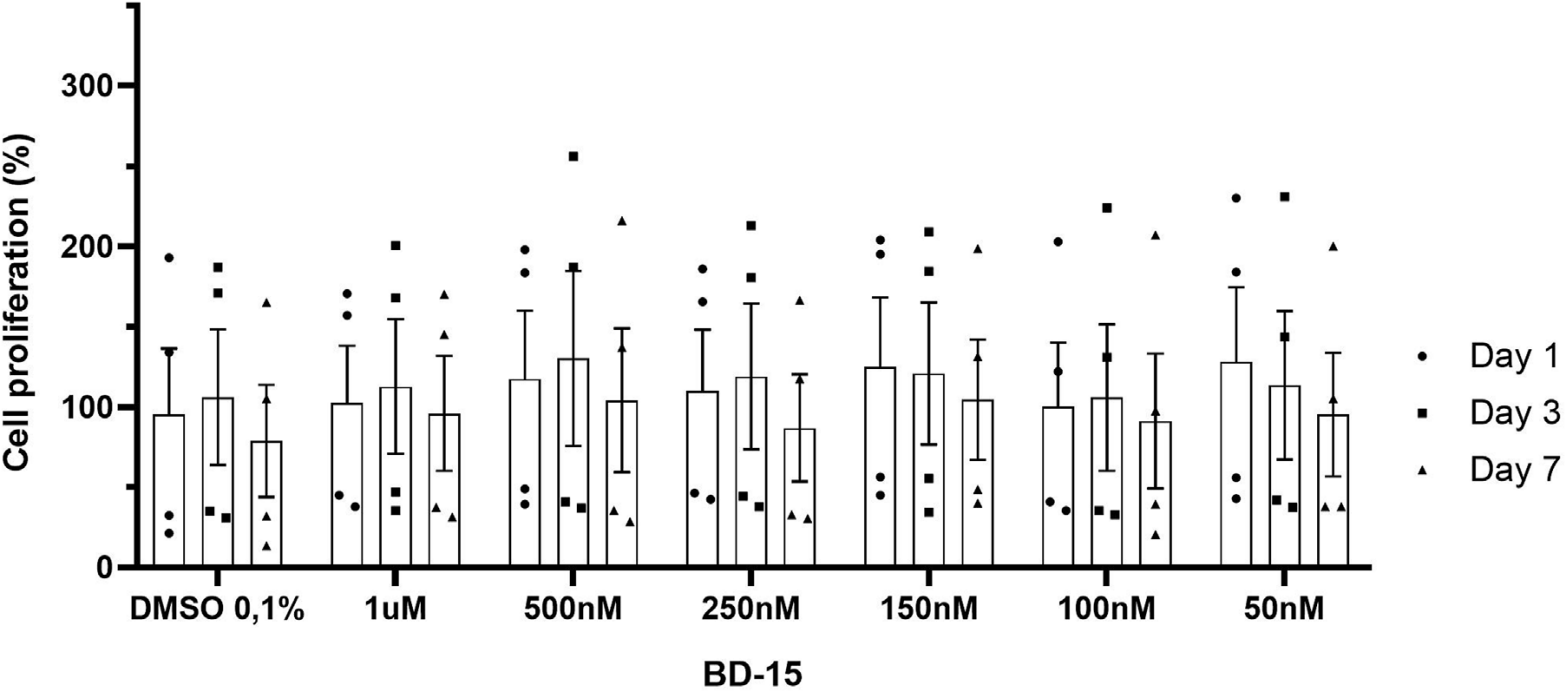
Evaluation of the proliferative effect of BD-15 vs. time). Data are presented as mean ± SEM. Statistical analysis was performed using one-way ANOVA with significance levels of *p < 0.05.

In our results, when we analyzed the effect of treatment time at the different concentrations, establishing the first day of treatment as the control, no statistically significant differences could be observed (1 day, 3 days, and 7 days). We analyzed the effect of time within each treatment, and there were no significant differences that showed that treatment with different concentrations of BD-15 altered the proliferation of these cells on the days analyzed (Figure 8).

**FIGURE 9:**
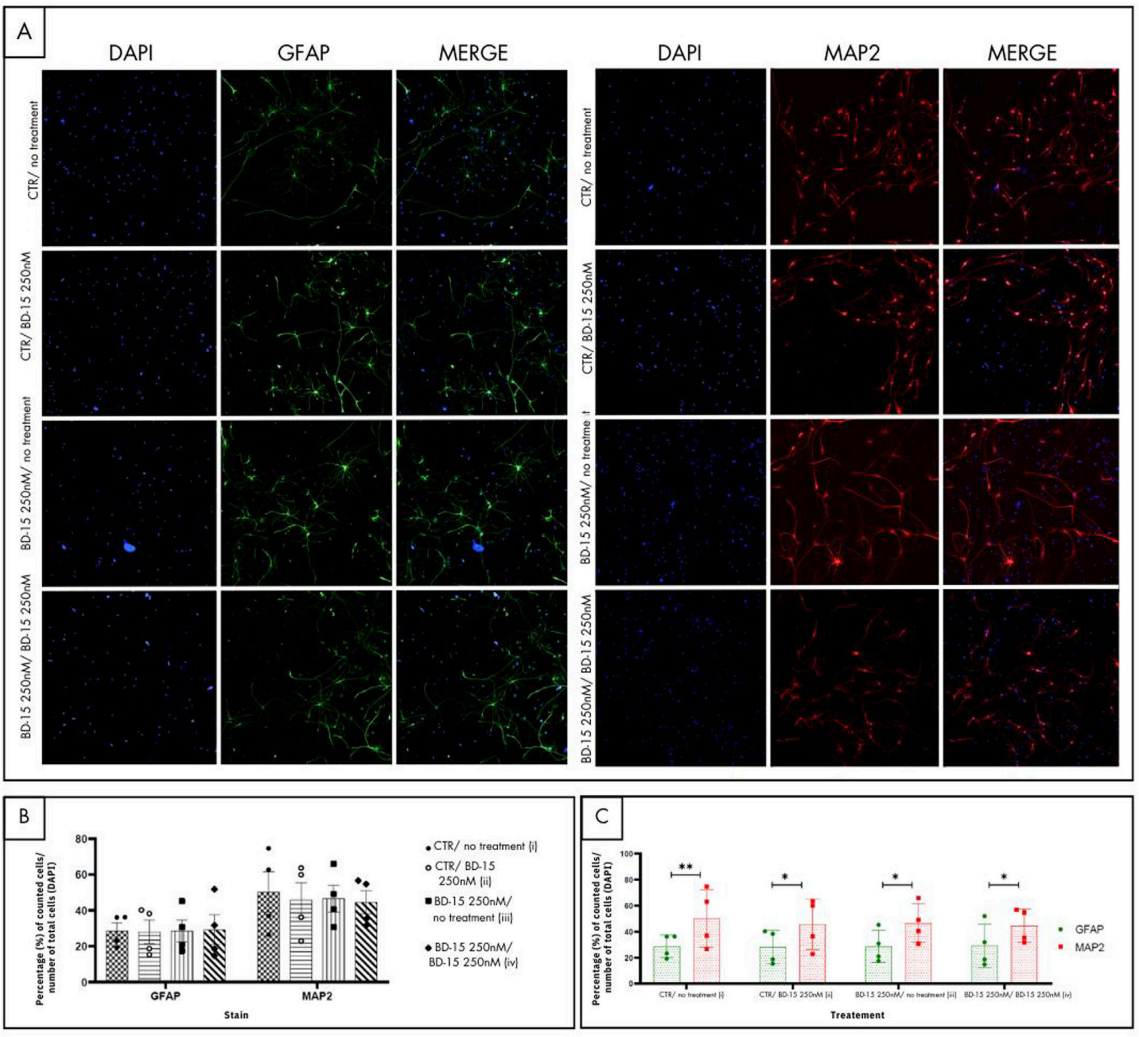
Immunofluorescence for cell differentiation. **(A)** The images in green show the astrocyte-labeled cells, and the images in red characterize the neuronal types. **(B)** and **(C)** For each photo, the proportions of GFAP and MAP2 in relation to the total number of cells (DAPI) were taken, and the average of these values was plotted on the graph. Statistical analysis was performed using one-way ANOVA with significance levels of *p < 0.05.

Immunofluorescence staining was carried out for microtubule-associated protein 2 (MAP2) and glial fibrillary acidic protein (GFAP) (Figure 9A), which are neuronal and astrocyte markers, respectively. The graph (Figure 9C) shows both markings for each treatment group. Firstly, an analysis was made of the number of cells marked for GFAP and MAP2 in each group. In the control group (i), a statistical difference (p = 0.0027) was detected between the number of cells marked by GFAP-labeled cells and the number of MAP2-labeled cells, showing that in our control culture of hippocampal neural precursor cells, there is a greater number of neuronal cells than astrocyte-labeled cells. It was also possible to observe statistical differences in the groups where the cells were treated with BD-150 250 nM at the time of plating (ii) (p = 0.0118), in the group in which the NPCs were placed in contact with BD-15 250 nM only before plating (iii) (p = 0.0101) and in the group in which the NPCS were treated at all times with BD-15 250 nM (iv) (p = 0.0282). From this analysis, it can be concluded that treatment with BD-15 250 nM at the different times does not interfere with the final ratio between the number of astrocytes and neurons.

Figure 9B illustrates the GFAP and MAP2 markings separately, comparing the number of astrocytes and neurons group by group. This graphical arrangement enables us to identify which group has a greater number of astrocytes and neurons. No statistical differences were identified when comparing the number of astrocytes (GFAP) of the control group with the other treatment groups, nor when groups were compared with each other. Similarly, no statistical differences were reported in the neuronal markings (MAP2) when the same analysis was carried out. The representation of the markings for each treatment group shows that the profile of BD-15 250 nM does not change, regardless of whether this substance was added after the cells were plated (ii), if the NPCs were under treatment with BD-15 250 nM only before experimentation (iii) or during the entire treatment period (iv).

## 8 Discussion

The present study investigated the properties of pre- and post-treatments with BD-15 on LPS-induced oxidative stress. BD-15 is a digoxin derivative that has shown potential as a therapy for brain disorders by decreasing oxidative stress in the cortex, cerebellum, hippocampus, and hypothalamus. Furthermore, this drug did not cause changes in heart and kidney tissues, demonstrating preferential specificity for the brain (Parreira et al., 2021).

Reduced locomotor activity is often associated with deficits in motor control and coordination, particularly in the frontal cortex and cerebellum, the most affected brain areas (Diamond, 2000; Kelly et al., 2020; Murphy et al., 2017). The results obtained demonstrate that acute post-treatment with BD-15 was not able to alter the locomotor capacity of the animals. Similarly, chronic treatment with BD-15 at different doses also did not alter locomotor activity in Wistar rats (Parreira et al., 2021). Locomotion is often impaired in neurological conditions, such as Parkinson’s disease, stroke, multiple sclerosis, and dementia (Allali et al., 2018). Our results demonstrated a reduction in locomotion in the LPS-treated group compared to the control group, which was reflected in a decrease in total and central quadrant crossings in the open field test.

Similar findings have been reported in Parkinson’s disease models, in which LPS-induced motor deficits are accompanied by increased oxidative stress (Kumar et al., 2023). In models of Huntington’s disease, animals subjected to LPS exhibit motor dysfunction, along with damage to the cerebellum, prefrontal cortex, and hippocampus (Kumar et al., 2023). Locomotor deficits have also been observed following LPS administration in other experimental models (Yang et al., 2020; Abdo Qaid et al., 2022; Beheshti et al., 2020). However, acute post-treatment with BD-15 did not interfere with LPS-induced locomotor capacity.

Our group previously reported a reversal of LPS-induced MDA levels in the hippocampus and cerebellum following ouabain treatment under acute experimental conditions (Garcia et al., 2018; Garcia et al., 2019). Additionally, chronic isolated BD-15 treatment lowered MDA levels in the prefrontal cortex (Parreira et al., 2021). However, in the present study, no significant effect was observed in the cerebellum after BD-15 treatment. Interestingly, we observed a significant reduction in protein carbonylation in the cerebellum, suggesting that post-treatment with BD-15 may reduce oxidative protein damage in this region, which may help explain the increase observed in the LPS + BD-15 group in the behavioral tests. Protein carbonylation, an oxidative modification resulting from the addition of aldehyde or ketone groups to amino acid residues, can impair enzyme function and contribute to neurological conditions, such as schizophrenia, Parkinson’s disease, amyotrophic lateral sclerosis, and Alzheimer’s disease (Akagawa, 2021; Martínez-Orgado et al., 2023; Ferrington et al., 2005; Floor and Wetzel, 1998; Koike et al., 2021; Shen et al., 2015). Similar findings have been reported in a study evaluating the neuroprotective effects of ouabain in the hippocampus of rats treated with LPS (Garcia et al., 2019).

Given the observed oxidative stress and protein damage, we evaluated the activity of GSH, an important antioxidant involved in neutralizing ROS and preventing cellular damage (Johnson et al., 2012; Liu et al., 2022; Lu, 2009). We observed a decrease in the GSH levels in the prefrontal cortex following LPS treatment. Reduced GSH activity can impair neurotransmitter function and affect memory and learning (Liu et al., 2022; Lu, 2009; Zeevalk et al., 2008). GSH is a key player in both direct antioxidant defense and regulation of glutathione-dependent enzymes (Ballatori et al., 2009; Tancheva et al., 2020). There is a well-established relationship between GSH levels and lipid peroxidation: diminished GSH levels are associated with increased lipid peroxidation (Kaur et al., 2020; Swarnkar et al., 2009). Our findings suggest that BD-15 posttreatment reversed LPS-induced oxidative damage in the cerebellum and cortex, as demonstrated by the increase in GSH levels. Previous studies by our group have also reported increased GSH activity following BD-15 treatment in the hippocampus and cortex, accompanied by a reduction in MDA levels (Parreira et al., 2021). Similarly, treatment with ouabain has shown a protective effect against LPS-induced oxidative stress in the hippocampal and cerebellar tissues (Garcia et al., 2018; Garcia et al., 2019).

To better assess the chronic effects of BD-15 on LPS-induced damage, Swiss albino mice were pre-treated with a dose of 0.56 mg/kg, which has been previously demonstrated to exhibit anti-inflammatory activity of ouabain (Leite et al., 2015; Rodrigues-Mascarenhas et al., 2011). This dose strategy is further supported by recent findings showing that another synthetic cardiotonic steroid γ-Benzylidene Digoxin 8 (BD-8), also demonstrated anti-inflammatory activity at the same dose (Ferreira et al., 2024). Pretreatment with BD-15 for three consecutive days significantly improved biochemical markers such as MDA, SOD, CAT and GST activity. Our results corroborate those of Parreira et al. (2021), who also observed improvements in oxidative damage in the cortex and hippocampus of Wistar rats chronically treated with BD-15 (Parreira et al., 2021). In addition, previous studies that treated mice chronically and observed increases in MDA levels, along with decreases in GSH, SOD, and CAT levels (Rodrigues et al., 2013; Taniguti et al., 2018; Jangra et al., 2014).

Considering the physiological differences between species, we evaluated the compound in both mice and rats to ensure a more comprehensive understanding of its effects. Although both are rodents, they exhibit significant distinctions that could influence the study’s outcomes. For instance, variations in neuron size and surface area (Vitale et al., 2023) may affect drug activity due to differences in receptor density. Additionally, structural differences in brain regions, such as the hippocampus (Routh et al., 2009), could impact the compound’s action within the central nervous system. Moreover, species-specific differences in NAK activity and expression levels may further influence the results. A study by Bao et al. (2020), demonstrated that sodium pump levels in mice were significantly higher than those observed in the rat brain cortex and non-cortical regions, highlighting key interspecies metabolic variations. These physiological disparities could ultimately affect the compound’s pharmacological profile and contribute to differences in research findings, emphasizing the importance of evaluating the compound in multiple.

Several studies have demonstrated that NKA activation plays a crucial role in cellular protection against oxidative stress across various pathological conditions. For instance, activation of NKA using the DRm217 antibody has been shown to protect cardiac cells from oxidative damage and mitochondrial dysfunction by stabilizing and increasing NKA activity. This protective effect is associated with the inhibition of Receptor Tyrosine Kinase (Src) phosphorylation, blockade of the NKA/ROS amplifier, and a reduction in calcium accumulation (Yan et al., 2017). Similarly, antioxidant therapies in rodent models of seizures and epilepsy have been shown to reduce oxidative stress, restore NKA function, and promote neuroprotection, leading to lower mortality rates and a reduced frequency of seizures (de Melo et al., 2023).

In line with these findings, our data demonstrated that chronic pretreatment with BD-15 exhibited a strong capacity to mitigate LPS-induced oxidative stress in different brain structures, in contrast to the more limited effects observed with acute post-treatment. This aligns with previous studies showing that a 3-day chronic treatment with BD-15 enhances the activity of the α3-NKA isoform in the cortex and hippocampus of rats (Parreira et al., 2021). Notably, BD-15 specifically targets the α3 isoform of NKA, whereas ouabain, another cardiotonic steroid (CTS), predominantly interacts with the α1 isoform, suggesting a more targeted mechanism of action for BD-15 in the central nervous system (Pessôa et al., 2018).

Interestingly, Jeremias et al. (2012) observed that sepsis reduces NKA activity in the cortex, and this effect can be reversed by antioxidant treatment, although it was not effective in the hippocampus (Jeremias et al., 2012). The literature extensively describes the interaction between CTS and NKA, with these compounds acting either as stimulators or inhibitors of NKA activity (Keenan et al., 2005; Shattock et al., 2015; Kometiani et al., 1998; Haas et al., 2000). Beyond their enzymatic effects, CTS also function as mediators of intracellular signaling, utilizing NKA as a receptor and signal transducer to initiate intracellular cascades (Bagrov et al., 2009; Zhang et al., 2008; Xie et al., 2013). When located in specialized membrane regions such as caveolae, NKA can interact with various signaling proteins, including Src and the Epidermal Growth Factor Receptor, leading to the activation of pathways such as Protein Kinase A, Protein Kinase C, and Phosphoinositide 3-Kinase. Depending on the specific stimulus, these interactions can either promote or suppress reactive oxygen species (ROS) production (Jensen et al., 2011; Elbaz et al., 2012).

These insights have driven growing interest in the development of synthetic CTS as potential therapeutic agents for neuroinflammatory and oxidative stress-related disorders. Our current findings suggest that chronic pretreatment with BD-15 may offer neuroprotection against oxidative stress, possibly by sustaining NKA activity during systemic inflammation. Although we did not directly measure NKA activity in our LPS model, existing evidence supporting BD-15’s ability to modulate NKA in other contexts strengthens the hypothesis of its therapeutic potential in conditions associated with LPS-induced oxidative stress.

Another hypothesis that could be involved in neuroprotection is neurogenesis in which our previous work confirmed that NPCs from the hippocampal dentate gyrus express α1 and α2 isoforms of NKA (Maria Orellana et al.) as it was demonstrated by Moseley et al. (2003) and Habiba et al. (2000). Moreover, we provided the first evidence that ouabain (OUA), a well-characterized CTS, promotes neurogenesis in NPCs through a mechanism involving increased levels of NeuroD2, BDNF, and NGF, alongside a transient reduction in Bdnf1 and a sustained decrease in NT3 expression. Given the neuroprotective effects observed with BD-15, we sought to determine whether it could similarly promote neurogenesis, as OUA does. (Maria Orellana et al.). To address this question, we evaluated the impact of BD-15 on NPC viability and proliferation. Our MTT and LDH assays confirmed that BD-15 is not neurotoxic, does not compromise the viability of NPCs derived from neonatal rats, and does not induce membrane lysis, indicating that it does not contribute to direct cell death. Additionally, across all tested concentrations, BD-15 did not alter cell proliferation, suggesting no significant effects on the NPC cell cycle. Finally, at 250 nM the concentration previously associated with the most robust neuroprotective effects—BD-15 did not enhance neuronal or astrocytic differentiation. These findings provide valuable insights into BD-15’s safety profile and its potential as a neuroprotective agent, while also distinguishing its effects from those of OUA in the context of neurogenesis.

## 9 Conclusion

Our findings demonstrate that BD-15 exhibits potential neuroprotective effects against LPS-induced oxidative stress, with notable differences between pre- and post-treatment approaches. While acute post-treatment with BD-15 was not sufficient to prevent LPS-induced locomotor impairment, it effectively reduced protein carbonylation in the cerebellum. In contrast, chronic pretreatment with BD-15 significantly improved oxidative stress markers across multiple brain regions, suggesting a more robust protective effect when administered preventively.

However, it is important to emphasize that these effects were observed in rodent models, specifically in Wistar rats and Swiss mice. Given interspecies differences in metabolism, receptor density, and Na⁺/K⁺-ATPase activity, caution is required when extrapolating these findings to other species, including humans. Future studies should focus on elucidating the precise molecular mechanisms underlying BD-15’s neuroprotective effects, as well as assessing its potential translational applications in neurological disorders characterized by oxidative stress and neuroinflammation. Additionally, further research should explore whether BD-15’s effects are region-specific within the brain or more broadly applicable across different neural structures.

By investigating both acute and chronic treatment paradigms, our study underscores the importance of treatment timing in mitigating oxidative stress. These insights contribute to the growing body of evidence supporting the role of cardiotonic steroids in neuroprotection and pave the way for future studies aimed at optimizing BD-15 as a potential therapeutic strategy.

## Data Availability

The raw data supporting the conclusions of this article will be made available by the authors, without undue reservation.
